# Rapid Extensively Drug‐Resistant (XDR) TB Diagnosis: An In‐House DNA Biochip for Drug Resistance Detection and Mutation Profiling of *Mycobacterium tuberculosis*

**DOI:** 10.1155/cjid/5518992

**Published:** 2026-07-27

**Authors:** Bharti Jain, Savita Kulkarni, Nawab Singh Baghel

**Affiliations:** ^1^ Radiation Medicine Centre, Bhabha Atomic Research Centre, Mumbai, Maharashtra, India, barc.ernet.in; ^2^ Homi Bhabha National Institute, Mumbai, Maharashtra, India, hbni.ac.in

**Keywords:** DNA biochip, drug resistance, extensively drug-resistant tuberculosis (XDR-TB), molecular diagnostics, multidrug-resistant TB, mutation detection, *Mycobacterium tuberculosis*

## Abstract

**Background:**

Emergence of multidrug‐resistant (MDR) and extensively drug‐resistant (XDR) *Mycobacterium tuberculosis (M. tuberculosis)* poses a major public health threat, especially in high burden countries. Current diagnostic modalities are slow, expensive, or inaccessible in low‐resource setting. We designed, fabricated, and validated an in‐house DNA biochip capable of detecting resistance‐associated mutations in key *M. tuberculosis* genes linked to resistance against first‐ (rifampicin and isoniazid) and second‐line (fluoroquinolones and second‐line injectables) antituberculosis drugs.

**Methods:**

The biochip allowed the detection of 20 drug resistance–determining mutations in the *rpoB, katG, inhA, gyrA, rrs,* and *eis* genes in the *M. tuberculosis* genome. Biochip consists of 33 probes spotted in duplicate including probe for *M. tuberculosis* detection. The biochip assay is based on the amplification of 7 fragments of the genome using two sets of multiplex PCRs. The biochip assay was optimized, and enhanced chemiluminescence was used for signal detection on biochip. Performance evaluation of the biochip was done using 175 clinical isolates. Culture‐based drug susceptibility test (DST) was used as the gold standard to compare biochip results, and sequencing was used to resolve the discordance.

**Results:**

Out of 59 culture sensitive isolates, 53 were sensitive to all drugs by biochip, while 6 isolates showed different mutations. The biochip showed high concordance with culture DST. The diagnostic sensitivity of the biochip assay for all the drugs ranged from 75% to 100%. The specificities of the biochip for all the 7 drugs were over 97%. The developed biochip demonstrated analytical sensitivity of 10^3^ genome copies per assay and showed high reproducibility, with intra‐assay coefficient of variation (CV) < 10% and interassay CV < 19% across all probes on the biochip.

**Conclusions:**

The biochip enables simultaneous analysis of multiple resistance‐associated mutation in a single assay providing a powerful tool for early detection, personalized therapy, and effective containment of both MDR and XDR‐TB. The biochip is useful for clinical microbiology studies and surveillance programs.

## 1. Introduction

Tuberculosis (TB) remains one of the most devastating infectious diseases worldwide. According to the WHO Global Tuberculosis Report 2024, 1.25 million people died of TB in 2023, making TB once again the leading cause of death from a single infectious disease, with mortality nearly twice that of HIV/AIDS [1]. Most patients are cured by 6 months of standardized first‐line therapy, but interrupted treatment or inappropriate antimicrobial use favors emergence of drug‐resistant (DR) strains. Multidrug‐resistant TB (MDR‐TB)—defined as *M. tuberculosis* resistant to at least isoniazid (INH) and rifampicin (RMP)—accounts for 3.2% of new and 16% of previously‐treated cases globally and treatment is longer, more toxic and less likely to succeed [1].

In January 2021, the WHO updated the definitions of pre‐extensively drug‐resistant TB (pre‐XDR‐TB) and extensively drug‐resistant TB (XDR‐TB) [2]. Pre‐XDR‐TB is now defined as MDR‐TB with additional resistance to any fluoroquinolone (FQ). XDR‐TB is defined as MDR‐TB with additional resistance to any FQ and at least one Group A drug (bedaquiline or linezolid). The earlier 2006 definition, which used resistance to FQs and second‐line injectable drugs (SLIDs; kanamycin [KAN], amikacin [AMI], and capreomycin [CAP]) as the criterion for XDR TB, has been superseded because the WHO‐recommended longer regimens for DR‐TB now center on bedaquiline, linezolid, levofloxacin or moxifloxacin, and clofazimine [3]. The WHO 2024 report estimates that 18% of MDR/RR‐TB cases now meet the pre‐XDR definition [1].

Current phenotypic drug susceptibility testing (DST) on solid medium can take up to 16–20 weeks to complete especially for XDR strains where DST is performed sequentially after culture and first‐line testing [4]. Delayed identification of pre‐XDR/XDR‐TB risks ongoing transmission and inappropriate empirical therapy. Molecular diagnostic approaches, including nucleic acid amplification tests (NAATs) and sequencing‐based methods, have significantly improved the speed of detection [5]. Several NAATs are available which include PCR‐based assays, hybridization‐based assays, and microarray‐based assays [6–12]. Widely used platforms such as GeneXpert MTB/RIF, Xpert MTB/XDR, and line probe assays (e.g., GenoType MTBDRplus, and MTBDRsl) are FDA‐approved and implemented in many settings but cover a fixed mutation panel and require parallel or sequential use of two cartridges or two strips for combined first‐ and second‐line resistance detection—at substantial per‐test cost [6–8]. Although, current diagnostic algorithms often employ a stepwise approach, wherein initial testing focuses on RMP or MDR‐TB detection, followed by second‐line DST when required and is appropriate in many settings, particularly where MDR/XDR prevalence is low, it increases turnaround time and risk of patient attrition in high‐burden settings or in cases of high clinical suspicion of drug resistance. WGS provides the most comprehensive resistance profile but its cost, infrastructure requirements, and bioinformatic complexity limit deployment in high‐burden settings. In this context, DNA biochip is a technology that is gaining interest for detecting both MDR‐TB and XDR‐TB strains, since it provides the possibility to investigate a high number of sites within different genes carrying mutations that lead to resistance.

DR in *M .tuberculosis* is attributed to random mutations in the *Mycobacterium* genome, which can be exploited to rapidly detect DR [13–15]. For RMP resistance, this includes common mutations in the *rpoB* gene (e.g., Codons 516, 526, 531, and 533), while INH resistance is associated with mutations in genes such as *katG* (e.g., Codon 315) and the *inhA* promoter region [16–19]. Resistance to FQ and SLID is most frequently associated with mutations in *gyrA* (Codons 90, 91, and 94) *and gyrB* genes for FQ resistance and in *rrs* (1401–1484 region) and *eis* genes for SLID resistance [20–25].

While commercial assays exist, their cost, limited mutation coverage, and requirement of significant laboratory infrastructure often restrict their use in many endemic regions. To address this gap, we developed an affordable in‐house DNA biochip capable of detecting major DR mutations for both first‐ and second‐line drugs in *M. tuberculosis.* Unlike conventional microarray platforms that rely on glass slides, this system integrates a polycarbonate track‐etched membrane (PC‐TEM)—an advanced microporous material offering a larger surface area for probe attachment and excellent spot morphology control as a solid support. Earlier work from our group led to the development of antibody chip for multianalyte detection of thyroid hormones enabling effective diagnosis of thyroid abnormalities, and DNA biochips for detecting mutations mediating first‐line/second‐line drug resistance in *M. tuberculosis* [26–30]. Although India’s National TB Elimination Program has transitioned to all‐oral regimens since 2024, the samples used in this study were retrospectively collected prior to this transition (2016–2019), when SLIDs such as KAN, AMI, and CAP were still part of standard treatment regimens. Therefore, inclusion of SLID‐associated resistance markers in the present biochip remains relevant for the clinical and programmatic context of the study period or in regions where SLID is still be used due to programmatic, economic, or supply‐chain constraints [31]. Specifically, 33 probes were integrated in the biochip to detect single nucleotide polymorphisms of 7 drug resistance‐related genes conferring RMP, INH, FQ, and SLIDs resistance. Development of XDR‐TB biochips is a logical advancement following MDR and second‐line resistance chips, aiming to provide a comprehensive, single‐step diagnostic tool for rapid detection of all major DR mutations in *M. tuberculosis*. Existing sequential testing approaches delay appropriate treatment and increase patient loss to follow‐up. Such an integrated platform directly supports WHO’s goal of universal DST and strengthens national TB control programs through faster, more reliable diagnosis of XDR‐TB. The aim of the present study is to describe the design and development of biochip for XDR detection and to evaluate, as a proof‐of‐concept analytical validation, its agreement with phenotypic DST.

## 2. Materials and Methods

### 2.1. *M. tuberculosis* Clinical Isolates and DNA Extraction

A panel of well‐characterized *M. tuberculosis* clinical isolates with known drug susceptibility profiles determined by phenotypic DST (including drug‐susceptible, mono/poly DR, MDR‐TB, pre‐XDR, and XDR‐TB) was used in this study. Samples were collected during 2016–2019 at TB hospital, Sewari, Mumbai when SLID‐based regimens were still in use under national TB control programs, for a different research objective. Genomic DNA was extracted from cultured *M. tuberculosis* isolates using a standardized DNA extraction protocol (cetyltrimethylammonium bromide [CTAB] method). DNA quality and quantity were assessed using nanodrop. The DNA samples were stored at −20°C.

### 2.2. Phenotypic Susceptibility Test

All clinical samples were decontaminated using the N‐acetyl‐L‐cysteine (NALC)–NaOH method with a final NaOH concentration of 1%. After centrifugation at 10,000 rpm for 20 min, the precipitate was resuspended in 1.5 mL of 0.5 M phosphate buffer (pH 6.8). All isolates were cultured on Löwenstein–Jensen (LJ) solid medium prior to DNA extraction. DST was carried out using the 1% proportion method on the LJ solid medium for RMP, INH, OFX, LVX, KAN, CAP, and AMI, after which the results were categorized as resistant or sensitive.

### 2.3. Multiplex PCR Amplification and Labeling

Target regions of the *16S rDNA*, *rpoB, katG, inhA* promoter, *gyrA, rrs,* and *eis* genes were amplified using multiplex PCR. Two sets multiplex PCR were optimized as all the genes could not be amplified using single multiplex PCR. One set (Set 1) of multiplex PCR included *16S rDNA, rpoB, katG*, and *inhA* promoter region and another set (Set 2) of asymmetric multiplex PCR included *gyrA, rrs*, and *eis* genes. Primers were designed to flank the mutation hotspots targeted by the probes on the biochip using primer blast software (https://www.ncbi.nlm.nih.gov/tools/primer-blast/) as described by us for some of genes previously [27, 30]. Complete list of primer used for both the multiplex PCR is given in Table 1. Reverse primer was labeled with biotin, and PCR amplification was performed using optimized conditions, as described in Table 1. Set 1 multiplex PCR tube contained 13 μL of PCR master mix (SuperPlex Premix, DSS Takara), 15 pmoles each of 16*S rDNA, rpoB, katG*, and *inhA* forward and 15 pmoles each of *16S rDNA, rpoB, katG*, and *inhA* reverse primers, and 3 μL of extracted DNA. Set 2 multiplex PCR tube contained 14 μL of PCR master mix (RR310A, DSS Takara) 15 pmoles of *gyrA, rrs,* and *eis* forward primers, 45 pmoles of *gyrA* reverse primer, and 15 pmoles each of *rrs* and *eis* reverse primers, and 2 μL extracted DNA sample.

**TABLE 1:** Primer sequences for multiplex PCR of first‐ and second‐line drug‐resistance gene targets.

Target region	Primer	Sequence (5′ ⟶ 3′)	PCR conditions
*Set 1: rpoB, katG, inhA, 16S rDNA*			
*rpoB*	rpoB‐F	TGGTCCGCTTGCACGAGGGTCAGA	3 min at 96°C; 2 cycles of 1 min at 96°C, 2 min at 72°C; 2 cycles of 1 min at 96°C, 1 min at 71°C, 1 min at 72°C; 2 cycles of 1 min at 96°C, 1 min at 70°C, 1 min at 72°C; 25 cycles of 1 min at 95°C, 1 min at 69°C, 1 min at 72°C; final 5 min at 72°C.
	rpoB‐R	CTCAGGGGTTTCGATCGGGCACAT	
*katG*	katG‐F	GAAACAGCGGCGCTGATCGT	
	katG‐R	GTTGTCCCATTTCGTCGGGG	
*inhA*	inhA‐F	CCTCGCTGCCCAGAAAGGGATCC	
	inhA‐R	CCGGGTTTCCTCCGGT	
*16S rDNA*	16S‐F	TCCTACGGGAGGCAGCAGT	
	16S‐R	GGACAACGCTCGCACCCTAC	

*Set 2: gyrA, rrs, eis (asymmetric multiplex)*			
*gyrA*	gyrA‐F	CGAACCGGTTGACATCGAGCAGGA	15 min at 95°C; 2 cycles of 1 min at 95°C, 35 s at 66°C, 35 s at 72°C; 2 cycles of 1 min at 95°C, 35 s at 65°C, 35 s at 72°C; 2 cycles of 1 min at 95°C, 35 s at 64°C, 35 s at 72°C; 2 cycles of 1 min at 95°C, 35 s at 63°C, 35 s at 72°C; 29 cycles of 1 min at 95°C, 35 s at 62°C, 35 s at 72°C; final 5 min at 72°C.
	gyrA‐R	CAGCATCTCCATCGCCAACG	
*rrs*	rrs‐F	CGTTCCCTTGTGGCCTGTGTGCAG	
	rrs‐R	GTTGGGGCGTTTTCGTGGTGC	
*eis*	eis‐F	GAAATTCGTCGCTGATTCTCGCAGTGGC	
	eis‐R	GCCGCGGCCAGTAGGAACATC	

*Note: rpoB, katG, inhA*, and *16S rDNA* were amplified together as Set 1; *gyrA, rrs*, and *eis* were amplified together as Set 2 under the conditions specified. Reverse primers were 5′‐biotinylated.

### 2.4. Biochip Design and Fabrication

**FIGURE 1:**
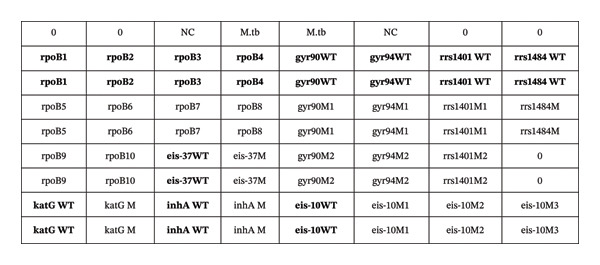
Layout of probes on the XDR M. tuberculosis detection biochip. The biochip carries 33 oligonucleotide probes spotted in duplicate (66 elements total) in an 8 × 8 grid on a polycarbonate track‐etched membrane. Wild‐type probes are shown in bold; mutant probes are in regular type. The M. tuberculosis‐specific 16S rDNA probe (M.tb) and the negative control (NC) are positioned in the top row to verify successful hybridization and the absence of nonspecific signal in every assay. NC = negative control; “0” = empty position without oligonucleotide.

Oligonucleotide probes are designed to be complementary to both the wild‐type (WT) and various mutant sequences within the target genes as described by us previously [27, 30]. To ensure efficient hybridization on the all the probes, secondary structures of oligonucleotides were estimated using OligoAnalyzer (Integrated DNA Technologies, http://eu.idtdna.com/analyzer/Applications/OligoAnalyzer/). The probes are typically designed to cover specific codons or regions known to harbor resistance‐conferring mutations, such as the *rpoB* hotspot region, codon 315 of *katG*, the promoter region of *inhA*, and the QRDR of *gyrA*, hotspot region of *rrs*, and *eis* promoter region. The biochip for the detection of mutations in *M. tuberculosis leading* to XDR consisted of 66 elements (33 oligonucleotides spotted in duplicate). The biochip included probe for *16S rDNA* region specific for *M. tuberculosis,* a probe for negative control, WT and mutated probes for detection of 6 mutations in *rpoB* gene, one mutation each in *katG and inhA*, 4 mutations each in *gyrA* and in *eis* promoter, and 3 mutations in *rrs* gene. The sequence of WT and mutated probes and the mutations targeted are given in Table 2. The biochip was fabricated on PC‐TEM. The designed oligonucleotide probes were synthesized with a 5′ amino‐C6‐modifier for covalent attachment to aldehyde‐activated PC‐TEM. Probe spotting was performed under controlled humidity and temperature at probe concentration of 10 µM in spotting buffer (3XSSC with 0.005% SDS). Following spotting, the biochips were washed and processed to block unreacted sites using 4% bovine serum albumin. The schematic layout of probe immobilization on the biochip is given in Figure 1.

**TABLE 2:** Sequences of the 33 oligonucleotide probes spotted on the XDR biochip.

Gene	Probe name	Mutation (amino‐acid change)	Sequence (5′ ⟶ 3′)
*16S rDNA*	M.tb	—	GTCCGGGTTCTCTCGG

*rpoB*	rpoB1	rpoB 514–520 WT	TTCATGGACCAGAACAACCCG
	rpoB2	rpoB 521–525 WT	CTGTCGGGGTTGACC
	rpoB3	rpoB 524–529 WT	TTGACCCACAAGCGCCGA
	rpoB4	rpoB 530–534 WT	CTGTCGGCGCTGGGG
	rpoB5	rpoB C531T (S531L)	CTG**TTG**GCGCTGGGG
	rpoB6	rpoB C531G (S531W)	CTG**TGG**GCGCTGGGG
	rpoB7	rpoB T533C (L533P)	GCG**CCG**GGGCCC
	rpoB8	rpoB C526T (H526Y)	TTGACC**TAC**AAGCGCCGA
	rpoB9	rpoB C526G (H526D)	TTGACC**GAC**AAGCGCCGA
	rpoB10	rpoB G516T (D516Y)	TTCATG**TAC**CAGAAC

*inhA*	inhA WT	inhA −15 WT	GCGGCGAGA**C**GATAGGT
	inhA M	inhA C‐15T	CGCGGCGAGA**T**GATAGG

*katG*	katG WT	katG WT (codon 315)	GATCA**G**CACCGGCATCGAGG
	katG M	katG G944C (S315T)	GATCA**C**CACCGGCATCGAGG

*gyrA*	gyrA 90WT	gyrA codon 90 WT	ACCACCCGCACGGCGAC**GCG**T
	gyrA 90M1	gyrA C269T (A90V)	ACCACCCGCACGGCGAC**GTG**T
	gyrA 90M2	gyrA T271C (S91P)	ACCCGCACGGCGACGCG**C**C
	gyrA 94WT	gyrA codon 94 WT	CGATCTAC**GAC**AGCCTGGT
	gyrA 94M1	gyrA A281C (D94A)	CGATCTAC**GCC**AGCCTGGT
	gyrA 94M2	gyrA A281G (D94G)	CGATCTAC**GGC**AGCCTGGTG

*rrs*	rrs1401 WT	rrs 1401 WT	ACCGCCCGTC**A**CGTCATGAA
	rrs 1401M1	rrs A1401G	ACCGCCCGTC**G**CGTCATGAA
	rrs 1401M2	rrs C1402A	ACCGCCCGTCA**A**GTCATGAA
	rrs1484 WT	rrs 1484 WT	CGGCGATTGGGAC**G**AAGTC
	rrs 1484M	rrs G1484T	CGGCGATTGGGAC**T**AAGTC

*eis (promoter)*	eis −10 WT	eis −10 WT	GCATATGCCA**C**A**G**TCGGATT
	eis −10M1	eis −10A	CATATGCCACA**A**TCGGATTCTG
	eis −10M2	eis −12T	GCATATGCCA**T**AGTCGGATTC
	eis −10M3	eis −14T	GGCATATGC**T**ACAGTCGGATT
	eis −37 WT	eis −37 WT	CGTAATATTCAC**G**TGCACGTAGC
	eis −37M	eis −37T	TAATATTCAC**T**TGCACGTAGCC

*Negative control*	NC	—	CTGGCAGCGCTGGGG

*Note:* Mutated nucleotides/codons are shown in bold. All probes were 5′‐amino‐C6 modified.

### 2.5. Hybridization and Washing

Hybridization mixture was prepared by adding 10 µL of amplified product from both multiplex PCR tubes (Set 1 and 2) to 200 μL of hybridization buffer (2× SSPE with 0.05% SDS). PCR products were denatured at 95°C and hybridized to the prepared DNA biochip under optimized hybridization conditions of 50 °C for 1 h in a hybridization oven. Following hybridization, the biochips were washed three times with (2× SSPE/0.25% SDS) for 10 min to remove nonspecifically bound DNA. The biochips were then incubated with streptavidin–horseradish peroxidase conjugate (1:4000 dilution in 2× SSPE/0.25% SDS) for 60 min at 42 °C to enable detection of biotin‐labeled PCR products hybridized to the probes. This was followed by multiple washing steps (3 washes with 2× SSPE/0.25% SDS, 5 min each) to remove unbound conjugate.

### 2.6. Signal Detection and Data Analysis

For signal development, enhanced chemiluminescence (ECL) substrate was added to the biochip and incubated for 1‐2 min. The emitted signal was captured using a gel documentation system (G:BOX, Syngene, UK). The presence or absence of specific mutations was determined by comparing the spot signal intensity of the mutant probes to that of the corresponding WT probes for each target region. The biochip results were compared with the known drug susceptibility profiles of the *M. tuberculosis* isolates determined by phenotypic DST.

### 2.7. Discordant Samples

To resolve the discordance between the culture DST and biochip results, sequencing was done. The following loci were extended by PCR, namely, *rpoB* (RMP), *katG* (INH), *inhA* (INH), *gyrA* (FQ), and *eis* and *rrs* (SLID), using the primers listed in Table 1. Sanger sequencing was performed to sequence the PCR products.

### 2.8. Statistical Analysis

Sensitivity, specificity, positive predictive value (PPV), and negative predictive value (NPV) of the biochip were calculated against phenotypic DST as the reference standard, with two‐sided 95% Wilson confidence intervals. Intermethod agreement was quantified by Cohen’s kappa (*κ*). Probe‐specific true‐positive (TP)/false‐positive (FP)/true‐negative (TN)/false‐negative (FN) and probe‐specific sensitivity and specificity were computed for analysis of probe‐specific performance.

### 2.9. Analytical Sensitivity

Analytical sensitivity (limit of detection, LoD) of the DNA biochip was determined by testing serial dilutions of genomic DNA from H37Rv (10^6^–125 genome copies). The diluted DNA was used for performing multiplex PCR and then hybridized to the biochip. The LoD of the biochip was defined as the highest dilution with reproducible hybridization signal that could be identified on all the probes and beyond which no signal was detected in one or more probes.

### 2.10. Precision

Intra‐assay reproducibility was evaluated by running 5 replicates of WT and MDR samples on five independently fabricated biochips within a single run, while interassay variation was evaluated across independent runs on different days with different biochip and multiplexed PCR batches and, where applicable, operators. Spot intensity was quantified using ImageJ software; the coefficient of variation (CV) was calculated for each probe along with concordance of mutations between the biochips.

## 3. Results

### 3.1. Characteristics of Study Isolates

Phenotypic DST of 175 *M. tuberculosis* culture isolates was performed using the proportion method on LJ solid medium. Sixty‐six samples were sensitive to all the drugs, 58 samples were mono resistant, 13 samples were polyresistant, 20 were MDR, 6 were pre‐XDR‐SLID, 10 were pre‐XDR‐FQ, and 2 were XDR by phenotypic DST.

### 3.2. Optimization of the High‐Grade Multiplex PCR Assay

For the efficient amplification of 7 segments of the *M. tuberculosis* genome, two sets of multiplex PCR were optimized. For *gyrA*, the concentration of the reverse adapter was higher than the forward to ensure preferential generation of single‐stranded fragments complementary to the oligonucleotides immobilized on the biochip. The lengths of the genomic segments amplified in the multiplex assay were in the range 178–540 bp, and the primers were arranged into two multiplex reaction mixtures (Set 1 and 2), as given in Table 1. We failed to obtain sensitive amplification of all 7 fragments in a single tube. A significant increase in multiplex PCR efficiency was achieved by the introduction of multiplex‐specific master mix. All 7 PCR products were obtained in (155/175) 88.5% of isolates. Seven drug‐sensitive isolates, 6 mono‐DR, 2 poly‐DR, 4 MDR, and 1 pre‐XDR‐FQ isolates did not give amplification for all the 7 genes and were excluded from the study. In total, 59 samples sensitive to all the drugs, 52 mono DR, 11 polydrug‐resistant, 16 MDR, 6 pre‐XDR‐SLID, 9 pre‐XDR‐FQ, and 2 XDR samples were analyzed by the biochip.

### 3.3. Biochip Analysis

**FIGURE 2:**
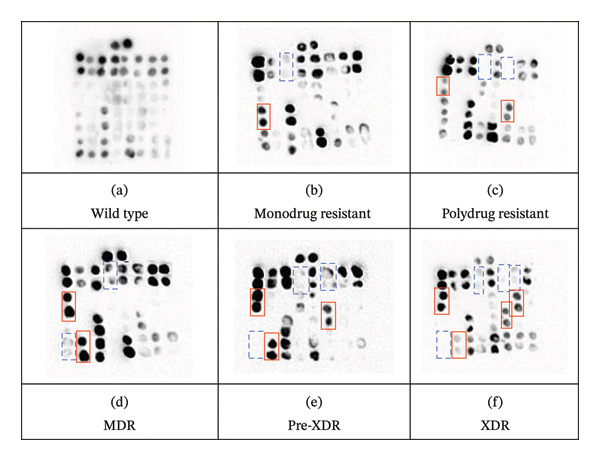
Analysis of *M. tuberculosis* drug resistance by hybridization on XDR biochip. Representative chemiluminescent images of biochips hybridized with (a) H37Rv wild‐type DNA—strong signal on all wild‐type probes and on the *M. tuberculosis* 16S rDNA probe; (b) a mono‐resistant (RMP) isolate carrying *rpoB* H526D—loss of signal on the *rpoB* 524–529 wild‐type probe (blue box) and gain of signal on the H526D mutant probe (red box); (c) a polydrug‐resistant (RMP + FQ) isolate carrying *rpoB* S531L and *gyrA* D94G—loss of wild‐type and gain of mutant signal at both loci; (d) an MDR isolate (*rpoB* S531L + *katG* S315T); (e) a Pre‐XDR isolate (*rpoB* S531L, *katG* S315T, and *gyrA* D94G); (f) an XDR isolate (carrying *rpoB* S531L, *katG* S315T, *gyrA* D94G, and *rrs* A1401G. Blue rectangles mark wild‐type probes that have lost signal due to the corresponding mutation; red rectangles mark mutant probes that have gained signal.

The mycobacterial target DNA loci responsible for the emergence of XDR were analyzed by hybridization on the developed XDR DNA biochip. The procedure consisted of two steps: (1) asymmetric multiplex PCR of the *gyrA, rrs* gene, and the *eis* promoter locus, (2) multiplex PCR for *rpoB, katG, inhA*, and *16S rDNA*, and (3) hybridization of amplified PCR products on the prepared DNA biochip. Biochip image was analyzed for the presence of spot signals on each biochip. The hybridization pattern corresponding to *M. tuberculosis* H37Rv WT DNA and various mutation obtained on different genes leading to specific DR is shown in Figure 2. The presence or absence of mutation can be detected by comparing the signal strength on WT and mutated probes for each gene.

### 3.4. Analytical Sensitivity

**FIGURE 3:**
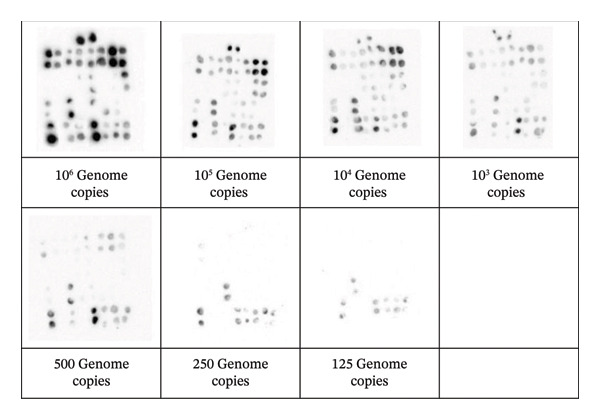
Limit‐of‐detection experiment. Serial dilutions of H37Rv genomic DNA (10^6^ to 125 genome copies per multiplex PCR) tested on the biochip. The wild‐type signal is reliable down to 10^3^ copies; at 500 genome copies *rpoB* WT probe signals and at 250 genome copies *rpoB, inhA, gyrA*, and *rrs* probe signals were not visible on the biochip.

The analytical sensitivity of this method was estimated by assaying serial dilutions of the purified genomic DNA of *M. tuberculosis* strain H37Rv. The hybridization results obtained with 10^3^ genomic equivalents were unambiguous. The analytical sensitivity of the final protocol was 10^3^ genomic equivalents per reaction (Figure 3). The next tested dilution (500 genomic equivalents per reaction) and further dilutions resulted in the absence of signals in some of the spotted probes.

### 3.5. Precision

The assay demonstrated good reproducibility for detection of major resistance‐associated mutations. Both intra‐assay and interassay variability analysis showed minimal variation in hybridization signal intensity among replicate samples processed within the same run and different runs, respectively. The intra‐assay %CV for all the probes on the biochip for the WT sample was < 12% and for MDR sample was < 9%. The inter‐assay %CV for all the probes for the WT sample was < 19% and for the MDR sample was < 16%. The %CV for all probes were within acceptable limits, indicating stable assay performance and reproducibility. Concordance of mutation detection between replicate experiments was 100% (Supporting Table S1).

### 3.6. Detection of Mutations and Correlation With Phenotypic Assays

Out of 155 samples, with amplification for all the gene segments, 59 were sensitive to all the drugs by culture DST. Of 59 samples that were susceptible to all drugs by culture DST, 53 were sensitive to all drugs by biochip, whereas 6 were mono‐DR. The presence of mutation in these isolates was confirmed by sequencing. The frequencies of different mutations detected by the biochip are listed in Tables 3 and 4. Biochip results are in agreement with sequencing results except for five isolates that did not show any mutation on the biochip. Mutational profile obtained from the biochip analysis and phenotypic drug resistance and the overall performance of the biochip test (sensitivity, specificity, NPV and PPV, and Cohen’s kappa [*κ*]) for individual drug are summarized in Table 3. The distribution of mutation combinations among polydrug‐resistant, MDR, pre‐XDR, and XDR isolates is presented in Table 4.

**TABLE 3:** Biochip analysis of the 155 successfully amplified clinical isolates: mutational profile, correlation with phenotypic DST, and overall biochip performance for each drug.

Drug	Gene	Detected mutation	R (DST)	S (DST)	Sensitivity % (95% CI)	Specificity % (95% CI)	PPV % (95% CI)	NPV % (95% CI)	*κ*
RMP	*rpoB*	S531L	23	1					
		H526Y	10	—					
		H526D	4	—					
		S531W	1	—					
		D516Y	1	—					
		Total with mutation	**39**	**1**	**95.1 (83.9–98.7)**	**99.1 (95.2–99.8)**	**97.5 (87.1–99.6)**	**98.3 (93.9–99.5)**	**0.95**
		Without mutation	**2**	**113**					

INH	*katG*	S315T	50	2					
	*inhA*	C‐15T	4	—					
	*katG + inhA*	S315T + C‐15T	3	—					
		Total with mutation	**57**	**2**	**95.0 (86.3–98.3)**	**97.9 (92.6–99.4)**	**96.6 (88.5–99.1)**	**96.9 (91.2–98.9)**	**0.93**
		Without mutation	**3**	**93**					

OFX	*gyrA*	D94G	5	1					
		D94A	1	—					
		A90V	1	—					
		Total with mutation	**7**	**1**	**87.5 (52.9–97.8)**	**99.3 (96.2–99.9)**	**87.5 (52.9–97.8)**	**99.3 (96.2–99.9)**	**0.87**
		Without mutation	**1**	**146**					

LVX	*gyrA*	D94G	7	—					
		A90V	1	—					
		Total with mutation	**8**	**0**	**88.9 (56.5–98.0)**	**100 (97.4–100)**	**100 (67.6–100)**	**99.3 (96.2–99.9)**	**0.94**
		Without mutation	**1**	**146**					

KAN/CAP/AMI	*rrs*	G1484T	4	—					
		C1402A	1	—					
		A1401G	3	2					
	*eis*	−14T	4	—					
		−12T	5	—					
	*rrs*	A1401G + G1484T	2	—					
		Total with mutation	**19**	**2**	**86.4 (66.7–95.3)**	**98.5 (94.7–99.6)**	**90.5 (71.1–97.3)**	**97.8 (93.6–99.2)**	**0.87**
		Without mutation	**3**	**131**					

CAP + AMI	*rrs*	A1401G + G1484T	1	—					
		Total with mutation	**1**	**0**	**100 (20.7–100)**	**100 (97.6–100)**	**100 (20.7–100)**	**100 (97.6–100)**	**1.00**
		Without mutation	**0**	**154**					

AMI	*rrs*	A1401G + G1484T	1	—					
		A1401G	2	—					
		Total with mutation	**3**	**0**	**75.0 (30.1–95.4)**	**100 (97.5–100)**	**100 (43.8–100)**	**99.3 (96.4–99.9)**	**0.85**
		Without mutation	**1**	**151**					

*Note:* Sensitivity, specificity, positive predictive value (PPV), and negative predictive value (NPV) are reported with 95% Wilson confidence intervals; agreement is reported as Cohen’s kappa (*κ*). RMP, rifampicin; INH, isoniazid; OFX, ofloxacin; LVX, levofloxacin; KAN, kanamycin; CAP, capreomycin; AMI, amikacin; R, resistant; S, susceptible; *κ*, Cohen’s kappa; DST, phenotypic drug susceptibility test.

Abbreviation: CI, confidence interval.

**TABLE 4:** Bio‐chip analysis of the clinical isolates providing detected mutations, its correlation with the phenotypic DST and sequencing for polydrug resistance, MDR, pre‐XDR, and XDR isolates.

Resistance (DST)	Gene(s)	Mutation(s) by biochip	*n*	Resolved as	Sequencing result
*Polydrug-resistant*					
RMP and OFX	*rpoB, gyrA*	S531L, D94G	1	—	—
	*rpoB*	S531L	1	RMP only	rpoB S531L; gyrA D94F

INH and KAN/CAP/AMI	*katG, eis*	S315T, −12T	2	—	—
	*katG*	S315T	1	INH only	katG S315T; rrs/eis no mutation
	*eis*	−14T	1	KAN/CAP/AMI only	eis −14T; katG/inhA no mutation
	*katG, rrs*	S315T, A1401G + G1484T	1	—	—

OFX and KAN/CAP/AMI	*gyrA, rrs*	D94G, A1401G + G1484T	1	—	—
INH and AMI	*katG, rrs*	S315T, A1401G	2	—	—
	*katG, rrs*	S315T, A1401G + G1484T	1	—	—

*MDR*					
RMP and INH	*rpoB, katG*	S531L, S315T	8	—	—
	*rpoB*	S531L	1	RMP only	rpoB S531L; katG/inhA no mutation
	*rpoB, katG, inhA*	H526Y, S315T, −15T	1	—	—
	*rpoB*	H526Y	1	RMP only	rpoB H526Y; katG/inhA no mutation
	*katG*	S315T	1	INH only	katG S315T; rpoB no mutation
	*rpoB, katG*	S531W, S315T	1	—	—
	*rpoB, katG*	D516Y, S315T	1	—	—
	*katG*	S315T	1	INH only	rpoB N518Y; katG S315T
	*katG*	S315T	1	INH only	rpoB S522P; katG S315T

*pre-XDR-FQ*					
INH, RMP, LVX	*rpoB, katG, gyrA*	H526Y, S315T, D94G	2	—	—
	*gyrA*	D94G	1	LVX only	gyrA D94G; rpoB/katG/inhA no mutation
	*rpoB, katG, gyrA*	S531L, S315T, D94G	1	—	—
	*rpoB, katG*	S531L, S315T	1	MDR	rpoB S531L; katG S315T; gyrA D94H
	*rpoB, katG*	H526Y, S315T	1	MDR	rpoB S531L; katG S315T; gyrA D94Y

INH, RMP, OFX	*rpoB, katG, gyrA*	H526D, S315T, D94G	2	—	—
	*rpoB, katG, gyrA*	S531L, S315T, A90V	1	—	—

*pre-XDR-SLID*					
INH, RMP, KAN/CAP/AMI	*rpoB, katG, eis*	S531L, S315T, −12T	1	KAN/CAP/AMI only	rpoB/katG/inhA no mutation; eis −12T
	*rpoB, katG, eis*	H526Y, S315T, −14T	1	—	—
	*rpoB, katG, rrs*	S531L, S315T, G1484T	2	—	—
	*rpoB, katG*	S531L, S315T	1	MDR	rpoB S531L; katG S315T; rrs G1454A
	*rpoB, katG*	H526D, S315T	1	MDR	rpoB H526D; katG S315T; rrs C1483T

*XDR*					
RMP, INH, LVX, KAN/CAP/AMI	*rpoB, katG, gyrA, eis*	H526Y, S315T, D94G, −14T	1	—	—
RMP, INH, OFX, KAN/CAP/AMI	*rpoB, katG, gyrA, rrs*	S531L, S315T, A1401G, D94G	1	—	—

*Note:* RMP, rifampicin; INH, isoniazid; OFX, ofloxacin; LVX, levofloxacin; FQ, fluoroquinolone; KAN, kanamycin; CAP, capreomycin; AMI, amikacin; DST, phenotypic drug susceptibility test.

Abbreviations: SLID, second‐line injectable drug; MDR, multidrug‐resistant; XDR, extensively drug‐resistant.

### 3.7. Drug Resistance

#### 3.7.1. RMP

Out of 155 samples that showed amplification for all the gene segments, 39 isolates were resistant to RMP by biochip among 41 culture‐resistant isolates. As expected, the *rpoB* S531L substitution was the most predominant mutation in resistant isolates (24/40) (60%). Of 39 isolates showing mutations by biochip, 8 were resistant to only RMP, 2 were polydrug‐resistant, 13 were MDR, and 14 were pre‐XDR and 2 were XDR. Two RMP‐resistant isolates were missed by the biochip. They had nucleotide insertion and showed signal in various WT and mutated probes and could not be appropriately analyzed by the biochip. Discrepant DST results were obtained, where one culture DST sensitive isolates showed S531L mutation. Sequencing analysis confirmed the presence of the mutation. The sensitivity and specificity for RMP detection by the biochip was 95.1% (83.9–98.7) and 99.1% (95.3–99.8), respectively, with a kappa (*κ*) value of 0.95 indicating almost perfect agreement with phenotypic DST (Table 3).

#### 3.7.2. INH

Of 155 isolates, 60 isolates were resistant to INH by culture DST, 57 of which were found resistant by biochip analysis (Table 3). *katG* S315T mutation was most commonly observed, shown by (52/59) (88.1%) of isolates. Of 57 isolates that were resistant by biochip, 50 had *katG* S315T mutation, 4 had *inhA*C‐15T mutation, and 3 had both *katG* S315T and *inhA*C‐15T mutations. Of these 57 isolates, 20 were resistant to only INH, 7 were polydrug‐resistant, 14 were MDR, 14 were pre‐XDR, and 2 were XDR. Three INH‐resistant isolates were missed by the biochip. One isolate had *katG* I335V mutation and 1 isolate had C‐10A in the *inhA* gene and were undetectable by biochip due to a lack of specific probe. One isolate had *katG* S315T mutation as confirmed by sequencing, and no signal was seen on both the WT and mutated probe on the biochip, and the isolate was missed by the biochip. Two isolates that were phenotypically sensitive showed *katG* S315T mutation by biochip. Sequencing analysis confirmed the presence of *katG* S315T mutation in one of these isolates. The sensitivity and specificity for INH detection by the biochip is 95.0% (86.3–98.3) and 97.9% (92.6–99.4), respectively, with *κ* = 0.93 (Table 3).

#### 3.7.3. FQ

Of the 17 isolates that were resistant to FQs by culture DST, 15 were resistant to FQs by biochip analysis. Biochip analysis shows that, 7 isolates resistant to OFX and 8 isolates resistant to LVX had *gyrA* mutation (Table 3). *GyrA* D94G was the most common shown by 5 isolates resistant to OFX and 7 isolates resistant to LVX. Other two isolates resistant to OFX had D94A and A90V mutation in *gyrA* gene, respectively. One isolate resistant to LVX had A90V mutation in *gyrA* gene. Out of 7 isolate that were resistant to OFX, 1 isolate was resistant to OFX only, 2 was polydrug resistant, 3 were pre‐XDR, and 1 was XDR. One isolate resistant to OFX was missed by the biochip. It had A90V mutation and did not show signal on the mutated probe and light signal on the WT probe. Out of 8 isolates that were resistant to LVX, 3 were resistant to LVX only, 4 were pre‐XDR, and 1 was XDR. The sensitivity and specificity for OFX detection by the biochip is 87.5% (52.9–97.8) and 99.3% (96.2–99.9), respectively; *κ* = 0.87 as *GyrA* D94G mutation was obtained on one of the OFX sensitive isolate. One isolates resistant to LVX were missed by the biochip due to unavailability of probe for *gyrA* G88C mutation, present in this isolate, as confirmed by sequencing. The sensitivity and specificity for LVX detection by the biochip is 88.9% (56.5–98.0) and 100% (97.4–100), respectively, with *κ* = 0.94 (Table 3).

#### 3.7.4. SLID

Twenty‐two isolates were resistant to KAN and CAP/AMI by culture DST as no isolate resistant solely to KAN was detected. Out of 19 isolates that were resistant to KAN and CAP/AMI by both culture DST and biochip, 4 had G1484T mutation, 1 had C1402A mutation, 3 had A1401G mutation, and 2 had A1401G and G1484T mutations in *rrs* gene. Four isolates had −14T and 5 isolates had −12T mutation in the *eis* promoter region. Out of 19 isolates that were resistant to KAN and CAP/AMI by culture DST, 8 showed resistance to KAN and CAP/AMI, 5 were polydrug resistant, 4 were pre‐XDR, and 2 were XDR. Mutations present in these isolates and their DR are given in Tables 3 and 4. Three isolates were missed by the biochip. As confirmed by sequencing, they had T1491C, A1499G, and G1454A mutations in the *rrs* gene that were missed by the biochip because the probes for these mutations were absent from the biochip. Two isolates sensitive to SLID by culture DST showed A1401G mutation in the *rrs* gene by biochip analysis. Sequencing analysis shows that one of these isolates had A1401G mutation and other had no mutation in *rrs* and *eis* gene. This may be due to wrong results obtained by the biochip. The sensitivity and specificity for KAN and CAP/AMI detection by the biochip is 86.4% (66.7–95.3) and 98.5% (94.7–99.6), respectively; *κ* = 0.87 (Table 3).

One isolate resistant to both CAP and AMI had a combination of A1401G and G1484T mutation. There were no FPs and negatives for CAP and AMI resistance. The sensitivity and specificity for CAP + AMI detection by the biochip was 100% (20.7–100) and 100% (97.6–100), respectively; *κ* = 1. Four isolates that were resistant to AMI by culture DST and had A1401G mutation in two isolates and A1401G and G1484T mutations in one isolate. One isolate was not detected by the biochip as it had C1402A mutation shown by sequencing, but no signal was obtained on the mutated probes, with only slight signal on the WT probe. The sensitivity and specificity for AMI detection by the biochip is 75% (30.1–95.4) and 100% (97.5–100) respectively; *κ* = 0.85. Out of 4 isolates that were resistant to AMI by culture DST, 1 was resistant to AMI only and three isolates were polydrug resistance with resistance to both AMI and INH.

#### 3.7.5. Polydrug Resistant

Eleven isolates were polydrug‐resistant by culture DST (Table 4). Two isolates were resistant to RMP and OFX. Five isolates were resistant to INH and KAN and AMI/CAP, one isolate was resistant to OFX and KAN and CAP/AMI, and 3 were resistant to AMI and INH. A discrepancy was observed when one isolate resistant to RMP and OFX showed S531L mutation in the *rpoB* gene but no mutation in the *gyrA* gene and was marked as RMP‐resistant by the biochip. This was due to the presence of a D94F mutation in the *gyrA* gene, probe for which was not present on the biochip. Mutations obtained in different genes by biochip analysis in these isolates are given in Table 4. Another discrepancy was observed where, two polydrug‐resistant isolates (resistant to INH as well as KAN and AMI/CAP) by culture DST had only *katG* S315T mutation and no mutation in *rrs* and *eis* gene in one isolate and was resistant to only INH. Other isolate had −14T *eis* mutation and was resistant to KAN and CAP/AMI and not to INH. This may be due to incorrect results obtained by the culture DST or due to the presence of mutations outside the amplified region or other genes like *tlyA,* involved in SLID resistance.

#### 3.7.6. MDR

In total, 16 isolates were MDR by culture DST. A combination of *rpoB* S531L and *katG* S315T was the most predominant mutation found in 50% (8/16) of the biochip‐resistant isolates. Two MDRs were missed by the biochip due to the absence of probes for rare mutations in *rpoB* gene (N518Y and S522P respectively) and were interpreted as resistant to only INH as they had *katG* G315C mutation also. Two isolates that were MDR by DST were marked as only RMP‐resistant by the biochip. One of them had *rpoB* S531L mutation. and the other had H526Y mutation and did not have any mutation in *katG* and *inhA* genes. This was due to incorrect results obtained by the culture DST or due to the presence of mutations outside the amplified region. Also, one isolate that was MDR by DST was marked as only INH‐resistant by the biochip, as it had *katG* S315T mutation again due to incorrect results obtained by culture DST as it has no mutation in *rpoB* gene (Table 4).

#### 3.7.7. Pre‐XDR

Fifteen isolates were pre‐XDR by culture DST (Table 4). Out of 15 pre‐XDR, 9 were pre‐XDR‐FQ and 6 were pre‐XDR SLID. A combination of *gyrA* D94G and *katG* S315T mutation along with different *rpoB* mutations is found on most of the pre‐XDR‐FQ with only 1 isolate had *gyrA* A90V mutation. For most of pre‐XDR‐SLID S531L mutation in *rpoB* gene and S315T mutation in *katG* gene along with different mutation on *rrs* and *eis* is seen predominantly. A discrepancy was observed where 2 pre‐XDR‐FQ isolates were shown as MDR by the biochip due the presence of D94Y and D94H mutations, respectively, in *gyrA* gene, probe for which was not present on the biochip. Also 2 pre‐XDR‐SLID were shown as MDR by the biochip as they had G1454A and C1483T mutation in *rrs* gene, respectively, which was undetectable by the biochip due to the absence of specific probes for these mutations. Also, one isolate that were pre‐XDR (resistant to INH, RMP, and LVX) by culture DST were marked as LVX‐resistant by the biochip as it was incorrectly identified as pre‐XDR by the culture DST. It had only D94G mutation in *gyrA* gene and no mutation in *rpoB, katG*, and *inhA* gene as confirmed by sequencing. One isolate that was resistant to INH, RMP, and KAN and CAP/AMI by culture and had only −12T mutation in the promoter region of *eis* gene and no mutation in *rpoB, katG*, and *inhA* gene as seen by sequencing. It was incorrectly identified as pre‐XDR by the culture DST or both isolates had mutations outside the amplified region.

#### 3.7.8. XDR

Two isolates that were XDR by phenotypic DST were correctly identified by the biochip. Mutations present in different genes in these isolates are given in Table 4.

### 3.8. Overall Concordance of Biochip With Phenotypic DST

Compared with conventional DST, the sensitivities of the biochip for detecting resistance to the 7 drugs ranged from 75% to 100%. The specificities of the biochip for all the 7 drugs were over 97% (Table 3). Cohen’s *κ* ranged from 0.85 to 1.00, denoting almost‐perfect agreement (Table 3). We also observed good agreement between the biochip (in the region covered by the probes) and the sequencing except for five isolates. Probe‐specific performance is provided in Supporting Table S2. Out of the 15 probes whose target mutation was represented in the cohort, and 11 had 100% sensitivity. *GyrA* A90V and *rrs* C1402A had lower sensitivities of 66.7% and 88.3%, respectively. Only one sample had *rrs* C1402A mutation that was missed by the biochip, thereby giving 0% sensitivity. Probe‐specific specificity was ≥ 97.8% for every probe, with only three probes (*rpoB* S531L, *katG* S315T, and *rrs* A1401G) yielding any FPs.

## 4. Discussion

The development of rapid and accurate diagnostic tool for DR detection in *M. tuberculosis* is a critical unmet need of the global public health. WHO has recommended the molecular test for detecting DR to both first‐ and second‐line drugs [32]. We have developed a DNA biochip that allows the identification of *M. tuberculosis* genomic substitutions that determine resistance to first‐line drugs (RMP and INH) and second‐line FQs and injection drugs (KAN, CAP, and AMI). SLID resistance markers were included on biochip as clinical isolates analyzed were retrospectively collected before the implementation of all‐oral regimens in India (2024), during which SLID were routinely used. DNA biochip consisted of 66 elements, containing probes for detecting *M. tuberculosis* and resistance to 7 anti‐TB drugs. The ability to detect mutations conferring resistance to both first‐ and second‐line drugs in a single assay offers significant advantages over conventional DST wherein results are available after 16–20 weeks, during which time many patients with MDR‐TB or XDR‐TB may succumb to the disease or transmit it to others or both. Conventional DST for second‐line drug testing is far less standardized than DST for first‐line drugs. Laboratories with a capacity for second‐line DST are even less common than those with capacity for first‐line DST. The developed in‐house DNA biochip demonstrates a promising approach for the rapid and simultaneous detection of mutations associated with both first‐ and second‐line drugs in *M. tuberculosis.*

Several molecular diagnostic platforms are commercially available for *M. tuberculosis* detection and DR profiling, including real‐time PCR (e.g., GeneXpert MTB/RIF and Xpert MTB/XDR test from cepheid), line probe assays (LPAs) (e.g., the GenoType MTBDR plus test and GenoType MTBDRsl from hain Lifesciences), and reverse hybridization microarrays (e.g., the BluePoint MtbDR array) [5]. Real‐time PCR assays (GeneXpert) provide high accuracy, a fast turnaround time, but it can detect resistance only to RMP and depends upon proprietary cartridges and instruments [33]. Xpert MTB/XDR version is available for detection of INH, FQ, and SLID resistance but it has high cost per cartridge, fixed mutation targets, and supply chain dependent [34]. Compared to the Xpert test, developed biochip can detect 7 drugs in a single assay. Besides, developed biochip can detect the exact mutations conferring DR and avoid the detection of false RMP resistance, such as L511P by the Xpert test. Both biochip and LPAs such as MTBDRplus and MTBDRsl utilize multiplex PCR followed by reverse hybridization on membranes strips. The developed biochip enables simultaneous detection of mutations associated with both first‐ and second‐line drug resistance within a single hybridization platform following multiplex amplification. Similar integrated approaches have been reported previously; however, the present system utilizes a PC‐TEM–based format that offers advantages in terms of probe immobilization, biochip fabrication simplicity, and potential cost‐effectiveness [12]. Thus, the present assay is an adequate tool to rapidly detect these point mutations that confer resistance to RMP, INH, OFX, MFX, KAN, AMI, and CAP.

Biochip assay was developed following sensitive amplification of 7 fragments of the *M. tuberculosis* genome in two parallel reactions. However, ∼ 11.4% (20/275) of isolates failed to yield amplification for all target genes and were excluded from the analysis, indicating a limitation in assay robustness. This can be attributed to low concentration of DNA (< 2 ng/μL in 11/20 isolates) or its degradation, as DNA used in this study was extracted from retrospective clinical isolates and stored for prolonged period at −30°C prior to analysis. Future optimization efforts will focus on improving assay robustness through the use of freshly extracted DNA and optimization of DNA extraction and storage protocols.

The biochip had high concordance with conventional DST assays. For RMP assays, the high concordance was expected since 95% of RMP‐resistant strains harbor mutations in the rifampicin‐resistance determining (RRDR) region of *rpoB* gene [35, 36]. Two RMP‐resistant and 2 MDR isolates were missed by the biochip. RMP‐resistant isolates was having nucleotide insertion and showed signal in various WT and mutated probes. MDR isolates had N518Y and S522P mutations in *rpoB* gene and were identified as INH‐resistant by the biochip. Also, one culture sensitive isolate showed *rpoB* S531L mutation by biochip and sequencing. Two MDR culture isolates were only RMP‐resistant by sequencing and biochip as they did not show any mutation in *katG* and *inhA* genes by sequencing.

Concerning INH resistance, three INH‐resistant isolates were missed by the biochip. Two had rare mutation in *katG (*I335V) and *inhA* (C‐10A) gene and probes for these mutations were absent from our biochip. One isolate had *katG* S315T mutation and was misinterpreted as INH‐sensitive by the biochip as no signal was obtained on mutated probe. Two culture sensitive isolates had *katG* S315T by biochip and sequencing. One culture MDR isolate was INH‐resistant by the biochip as it was having only *katG* mutation and no mutation in *rpoB* gene as shown by biochip and sequencing. Two culture MDR isolates were deciphered as INH‐resistant by the biochip as they had rare mutation N518Y and S522P in *rpoB* gene, probe for which was not present on the biochip.

Regarding FQs, one isolate resistant to OFX was missed by the biochip as it had A90V mutation (shown by sequencing) and did not show signal on the mutated probe. One isolates resistant to LVX were missed by the biochip due to the presence of *gyrA* G88C mutation probe for which was not present on the biochip. One polydrug‐resistant isolate (resistant to RMP and LVX) by culture DST was shown as RMP‐resistant by the biochip as it also had D94F mutation in *gyrA* gene that was missed by the biochip due to the absence of probe for this mutation. Also, two pre‐XDR‐FQ isolates were shown as MDR by the biochip due the absence of probes for rare mutations in *gyrA* (D94Y and D94H).

Regarding SLIDs, three isolates resistant to KAN and CAP/AMI were identified as susceptible by the biochip. They had T1491C, A1499G, and G1454A mutations in *rrs* gene that was missed by the biochip. Two phenotypic susceptible isolates had *rrs* A1401G mutation as shown by the biochip, but sequencing analysis showed the presence of *rrs* A1401G mutation in one of the isolates and other isolates had no mutations in *rrs* or *eis* gene. This was due to misinterpretation of results by the biochip for one isolate showing wrong signal on the mutated probe. One isolate resistant to AMI was missed by the biochip as it had C1402A mutation as confirmed by sequencing but no signal was seen on the mutated probe. Two pre‐XDR‐SLID isolates were shown as MDR by the biochip due to the absence of probes for rare mutations in the *rrs* gene (G1454A and C1483T).

Although most of the gene mutations could be accurately obtained by comparing hybridization signal levels detected using both the WT and mutant type probes, ambiguous hybridization signals were observed for certain mutations. Probes showing suboptimal performance, especially *GyrA* A90V, *rrs* A1401G, and *rrs* C1402A, having lower sensitivity of mutation detection and *rpoB* S531L, *katG* S315T, and *rrs* A1401G showing some FPs resulting in < 100% specificities, may lead to misinterpretation. This indicates that further optimization of probe design and probe length is required to prevent visual misinterpretation.

The analytical sensitivity of the biochip was 10^3^ genome equivalents per reaction. Although the assay demonstrated slightly lower analytical sensitivity compared with cartridge‐based real‐time PCR systems such as GeneXpert (112.6 CFU/mL), its performance is comparable to hybridization‐based line probe assays, particularly considering its multiplex mutation detection capability across multiple resistance‐associated loci. The assay also showed high reproducibility with minimal variation in signal intensity between and within replicate experiments. Intra‐assay variability analysis showed < 10% CV for MDR samples and < 12% for WT samples, while interassay variability remained < 19% across all probes. Moreover, mutation detection concordance between replicate experiments was 100%, indicating stable hybridization performance and reliable mutation detection across different runs, PCR batches, and independently fabricated biochips.

Overall, as compared to culture DST, the biochip demonstrated good diagnostic sensitivity and specificity for detecting resistance to the 7 individual drugs. The sensitivities of the biochip for detecting resistance to the 7 drugs ranged from 75% to 100%. The sensitivity was higher for first‐line drugs (∼95%), whereas sensitivities for second‐line drugs ranged from 75% to 100%. The observed sensitivity for second‐line drugs, although encouraging, is based on a limited number of resistant isolates in certain resistance subgroups, particularly XDR‐TB (*n* = 2), pre‐XDR‐SLID (*n* = 6), and pre‐XDR‐FQ (*n* = 9). This reflects the lower prevalence of these resistance patterns in the study setting due to the retrospective nature of sample selection. Further validation using larger and more diverse cohorts, including a higher number of XDR and pre‐XDR isolates, is required to establish the robustness of the assay for these clinically important categories. The specificities of the biochip for all the 7 drugs were over 97% (Table 3). We also observed good agreement between the biochip (in the region covered by the probes) except for 5 (3.2%) isolates.

A key limitation of the biochip is its reliance on predefined mutation hotspots, which may result in false‐negative results when resistance is mediated by rare or emerging mutations outside the targeted regions. This was evident in several discordant isolates in the present study (e.g., mutations in *gyrA, rrs, katG,* and *inhA* not covered by the probe set). However, the modular design of the biochip platform allows for iterative updating of probe content without optimizing the multiplex PCR by adding new probes. New probes can be incorporated based on mutations identified through sequencing of discordant isolates *(gyrA* G88C, D94Y, D94H, D94F; *rrs* T1491C, A1499G, G1454A, and C1483T; *katG* I335V; *inhA* C‐10A), as well as data from global and region‐specific resistance surveillance studies and curated mutation databases [37]. Such an adaptive approach would enable continuous refinement of the assay, improving mutation coverage and diagnostic performance over time. Integration with next‐generation sequencing data could further support evidence‐based probe selection in future versions of the biochip.

The rapid turnaround time of the biochip assay, compared to the weeks required for phenotypic DST, could significantly impact clinical decision‐making, allowing for timely initiation of appropriate treatment regimens and improved patient outcomes. Furthermore, the multiplex nature of the biochip assay can provide a comprehensive DR profile, which is crucial for managing complex cases of XDR‐TB. This biochip assay can be considered a “rule‐in” test for MDR, second‐line drug resistance, although it cannot reliably rule out MDR/XDR‐TB when no genetic mutations are detected.

Commercially available assays such as GeneXpert and LPA require expensive instruments and/or high‐cost cartridges. In contrast, the cost of the biochip is approximately Rs. 800–1000, almost half the price of one GeneXpert MTB/RIF test which costs Rs. 2000–3000 in India excluding the cost of the instrument. For detecting XDR additional testing on GeneXpert MTB/XDR is required that cost ∼ Rs. 4000 in India. LPA such as MTBDRplus and MTBDRsl are priced at ∼ Rs. 2000 and 5000, respectively, excluding the equipment cost [38]. Besides this, no other expensive equipment is required for biochip analysis. The only instrument used in biochip analysis is a small shaker incubator for performing the hybridization process, and gel documentation system for capturing the chemiluminescence signals. Thus, the in‐house development of the biochip offers the potential for cost‐effectiveness, particularly in resource‐limited settings where the burden of DR‐TB is high.

However, several limitations of this study need to be addressed. A key limitation of the present study is that the assay was evaluated using DNA extracted from cultured isolates rather than direct clinical specimens. While this approach enabled robust validation against phenotypic DST, it does not fully exploit the potential advantage of molecular diagnostics in reducing turnaround time. Culture‐based isolation of *M. tuberculosis* can take several weeks, thereby delaying diagnosis. Future work will focus on adapting and validating the biochip for direct application on clinical samples such as sputum, which would significantly enhance its clinical utility by enabling rapid detection of drug resistance. Nevertheless, evaluation using cultured isolates is a standard initial step in assay development to ensure analytical accuracy before transitioning to direct specimen testing.

For successful implementation in routine TB diagnostic programs, workflow, and operational considerations are critical. The current assay is designed for use in laboratories with the existing molecular diagnostic infrastructure. Handling of live *M. tuberculosis* cultures requires Biosafety Level 3 (BSL‐3) facilities; however, following DNA extraction and inactivation, subsequent steps including PCR amplification and hybridization can be performed in BSL‐2 laboratory settings. The complexity of the biochip fabrication and hybridization process requires specialized equipment and expertise which may limit its use in peripheral laboratories but is feasible in reference and intermediate‐level laboratories. Simplification and automation of the workflow could enhance its feasibility for routine diagnostic use.

## 5. Conclusion

In conclusion, the developed in‐house DNA biochip provides a rapid and potentially cost‐effective platform for the simultaneous detection of key mutations associated with XDR in *M. tuberculosis.* In the proof‐of‐concept analytical validation, developed biochip demonstrate high sensitivity (75%–100%) and specificity (> 97%) in identifying resistance‐conferring mutations. Further research and optimization are warranted to fully establish its clinical utility as a valuable tool for the diagnosis and management of XDR‐TB, particularly in resource‐limited settings.

## Author Contributions

Conceptualization: Bharti Jain and Savita Kulkarni; methodology: Bharti Jain and Savita Kulkarni; formal analysis and investigation: Bharti Jain; writing–original draft preparation: Bharti Jain; writing–review and editing: Bharti Jain and Savita Kulkarni; supervision: Nawab Singh Baghel.

## Funding

This study was funded by BARC.

## Disclosure

All authors read and approved the final manuscript.

## Ethics Statement

This study was conducted in accordance with the Declaration of Helsinki, approved by the Institutional ethics committee of Bhabha Atomic Research Centre. Due to the retrospective nature of the study, and anonymization of patients’ data and information, no informed consent was required from the participants.

## Conflicts of Interest

The authors declare no conflicts of interest.

## Supporting Information

Additional supporting information can be found online in the Supporting Information section.

## Supporting information


**Supporting Information** Supporting Table S1: Intra‐ and interassay reproducibility. Supporting Table S2: Probe‐specific performance of the XDR biochip.

## Data Availability

All data generated or analyzed during this study are included in this published article. Additional datasets are available from the corresponding author on reasonable request.
