# Supplementary material for: Rapid Extensively Drug‐Resistant (XDR) TB Diagnosis: An In‐House DNA Biochip for Drug Resistance Detection and Mutation Profiling of Mycobacterium tuberculosis
Source: Can J Infect Dis Med Microbiol. 2026 Jul 27;2026:5518992. doi: 10.1155/cjid/5518992 (PMC13402939; doi:10.1155/cjid/5518992)
Supplement: Supplementary file 1 — Supporting Information Supporting Table S1: Intra‐ and interassay reproducibility. Supporting Table S2: Probe‐specific performance of the XDR biochip. [file CJID-2026-5518992-s001.docx]

**Supplementary Table S1.** Intra- and inter-assay reproducibility.

| **Reference isolate** | **Replicates** | **Intra-assay CV (%)** | **Inter-assay CV (%)** | **Concordance of mutation detection** |
| --- | --- | --- | --- | --- |
| WT Isolate | n = 5 (intra) / 5 (inter) | <9% | <19% | 100% |
| MDR isolate (rpoB S531L + katG S315T) | n = 5 (intra) / 5 (inter) | <10% | <16% | 100% |
|  |  |  |  |  |

A wild type and MDR isolate were tested on five independently fabricated biochips within a single run (intra-assay) and on five independently fabricated biochips and multiplexed PCR batches on five separate days (inter-assay). Spot intensity was quantified with ImageJ; the coefficient of variation (CV) was calculated for each probe. Concordance of mutation detection reflects whether the same genes was called wild-type / mutant across all replicates.

*CV, coefficient of variation; WT, wild type; MDR, multidrug resistant;*

**Supplementary Table S2.** Probe-specific performance of the XDR biochip.

| **Gene** | **Probe** | **Mutation** | **TP** | **FP** | **TN** | **FN** | **Sensitivity % (95% CI)** | **Specificity % (95% CI)** |
| --- | --- | --- | --- | --- | --- | --- | --- | --- |
| ***rpoB*** | rpoB1–4 | RRDR WT (514–534) | — | — | — | — | — | — |
| ***rpoB*** | rpoB5 | S531L (C531T) | 24 | 1 | 130 | 0 | 100.0 (86.2–100.0) | 99.2 (95.8–99.9) |
| ***rpoB*** | rpoB6 | S531W (C531G) | 1 | 0 | 154 | 0 | 100.0 (20.7–100.0) | 100.0 (97.6–100.0) |
| ***rpoB*** | rpoB7 | L533P (T533C) | 0 | 0 | 155 | 0 | n.a. | 100.0 (97.6–100.0) |
| ***rpoB*** | rpoB8 | H526Y (C526T) | 10 | 0 | 145 | 0 | 100.0 (72.2–100.0) | 100.0 (97.4–100.0) |
| ***rpoB*** | rpoB9 | H526D (C526G) | 4 | 0 | 151 | 0 | 100.0 (51.0–100.0) | 100.0 (97.5–100.0) |
| ***rpoB*** | rpoB10 | D516Y (G516T) | 1 | 0 | 154 | 0 | 100.0 (20.7–100.0) | 100.0 (97.6–100.0) |
| ***katG*** | katG M | S315T (G944C) | 53 | 2 | 97 | 1 | 98.1 (90.2–99.7) | 98.0 (92.9–99.4) |
| ***inhA*** | inhA M | C-15T | 7 | 0 | 148 | 0 | 100.0 (64.6–100.0) | 100.0 (97.5–100.0) |
| ***gyrA*** | gyrA 90M1 | A90V (C269T) | 2 | 0 | 152 | 1 | 66.7 (20.8–93.9) | 100.0 (97.5–100.0) |
| ***gyrA*** | gyrA 90M2 | S91P (T271C) | 0 | 0 | 155 | 0 | n.a. | 100.0 (97.6–100.0) |
| ***gyrA*** | gyrA 94M1 | D94A (A281C) | 1 | 0 | 154 | 0 | 100.0 (20.7–100.0) | 100.0 (97.6–100.0) |
| ***gyrA*** | gyrA 94M2 | D94G (A281G) | 12 | 0 | 143 | 0 | 100.0 (75.7–100.0) | 100.0 (97.4–100.0) |
| ***rrs*** | rrs 1401M1 | A1401G | 5 | 1 | 148 | 1 | 83.3 (43.6–97.0) | 99.3 (96.3–99.9) |
| ***rrs*** | rrs 1401M2 | C1402A | 0 | 0 | 154 | 1 | 0.0 (0.0–79.3) | 100.0 (97.6–100.0) |
| ***rrs*** | rrs 1484M | G1484T | 6 | 0 | 149 | 0 | 100.0 (61.0–100.0) | 100.0 (97.5–100.0) |
| ***eis*** | eis −10M1 | −10A | 0 | 0 | 155 | 0 | n.a. | 100.0 (97.6–100.0) |
| ***eis*** | eis −10M2 | −12T | 5 | 0 | 150 | 0 | 100.0 (56.6–100.0) | 100.0 (97.5–100.0) |
| ***eis*** | eis −10M3 | −14T | 4 | 0 | 151 | 0 | 100.0 (51.0–100.0) | 100.0 (97.5–100.0) |
| ***eis*** | eis −37M | −37T | 0 | 0 | 155 | 0 | n.a. | 100.0 (97.6–100.0) |

*TP, true positive; FP, false positive; TN, true negative; FN, false negative. Sensitivity is reported only when the relevant mutation was represented in the cohort; specificity is reported for all probes. n.a., not applicable (no isolate carried this mutation). Probe-specific sensitivity for some probes (rpoB6, B10, gyrA 94M1) is 100% based on a single TP; the confidence interval is therefore wide.*
